# Supplementary material for: Proteomic Subtyping of Alzheimer’s Disease CSF links Blood-Brain Barrier Dysfunction to Reduced levels of Tau and Synaptic Biomarkers
Source: bioRxiv. 2025 Mar 15:2025.03.14.643332. Preprint. [Version 1] doi: 10.1101/2025.03.14.643332 (PMC11952530; doi:10.1101/2025.03.14.643332)

bioRxiv preprint doi: https://doi.org/10.1101/2025.03.14.643332; this version posted March 15, 2025. The copyright holder for this preprint (which was not certified by peer review) is the author/funder, who has granted bioRxiv a license to display the preprint in perpetuity. It is made available under aCC-BY 4.0 International license.

Supplemental Figure 1: Variance partition analysis reveals that proteins associated with AD diagnosis differ based on race and sex. (A) Variance partition analysis was performed to determine to what magnitude the factors of diagnosis, age, race, and sex contributed to variations in cohort protein abundance, and to confirm minimal contributions to data variance due to cohort or TMT batch. The top 5 proteins with the greatest variance in abundance attributable to each factor are noted. **(B)** The top 20 proteins with the greatest variance in abundance across cases due to AD diagnosis also have notable contributions to their variance attributable to the sex, race, and age of the participant. Bolded proteins labels indicate proteins where the combined variance due to race and sex is greater than 10% of that due to diagnosis.

Supplemental Figure 2: Module correlations within the co-expression network. (A) Pearson correlations and p values corresponding to protein modules within the network demonstrating positive (red) or negative (blue) associations. **(B)** Eigenprotein values from Module 10 broken out by participant demographic. Significance assessed by 1-way ANOVA, points outside of 3 standard deviations for each subtype were not plotted.

Supplemental Figure 3: Influence of Diagnosis, Sex, and Race on Protein Variance and Module Abundance in the Emory Cohort. (A) Variance partition analysis of the Emory cohort regressed for all variables except diagnosis (**left**), sex (**center**) and race (**right**) demonstrate the effective minimization of other sources of variance. Plots are annotated with the top 5 proteins with the greatest variance in abundance due to each factor. **(B)** Volcano plots displaying the log2-fold change of protein abundance plotted against the FDR corrected 1-Way ANOVA p value, demonstrate the differential protein abundance of the preserved factors of diagnosis (**left**), sex (**center**) and race (**right**) in the absence of other variables. Proteins are annotated by module color, illustrating the relationships between modules and demographic factors. **(C)** A fractional breakdown of the proteins in each module that have significant differential abundance based on the selected factors of diagnosis (**left,** blue: control, red: AD), sex (**center,** blue: male, red: female) and race (**right,** blue: AA, red: NHW), highlighting the fundamental influence of diagnosis and demographics on module abundance, even in the absence of the other sources of variance.

Supplemental Figure 4: Network Module Eigenprotein Levels Across UMAP Subtypes in the Swiss Replication Cohort. Additional network module eigenprotein boxplots for the Swiss Replication cohort, broken

bioRxiv preprint doi: https://doi.org/10.1101/2025.03.14.643332; this version posted March 15, 2025. The copyright holder for this preprint (which was not certified by peer review) is the author/funder, who has granted bioRxiv a license to display the preprint in perpetuity. It is made available under aCC-BY 4.0 International license.

out by assigned UMAP subtype in order of module relatedness. Significance was assessed by 1-way ANOVA, points outside of 3 standard deviations for each subtype were not plotted.

Supplemental Figure 5: Correlation between the Emory and Alzheimer's Center Amsterdam Subtypes. (A) A heatmap showing the correlation between the z-scored mean protein abundances of participants in the Emory and Alzheimer's Center Amsterdam (ACA) proteomic subtypes. Subtypes with strong positive (red) or negative (blue) bicor values between the cohorts indicate relatedness (*p≤0.05; **p≤0.01; ***p≤0.001 ****p≤0.0001). **(B)** Corresponding immunoassay CSF Tau levels from each ACA subtype, where all participants have been diagnosed with AD. ACA subtypes that were highly correlated with Emory Subtype 3 (ACA Subtype 4/Choroid Plexus Dysfunction, ACA Subtype 5/Blood Brain Barrier Dysfunction) also had the lowest levels of CSF Tau.

Supplemental Figure 6: Module 1 members Plasminogen and ADAM10 are capable of cleaving recombinant tau. **(A)** Western blot demonstrating that plasminogen is capable of cleaving biotinylated recombinant tau (~64 kDa) over 120 minutes, a process which is partially inhibited by tranexamic acid, as is ADAM10 (**B**).

Supplemental Figure 7: Comparison of AD Pathology-Associated CSF Modules Between Subtype 6 and Subtype 3 AD Samples. A breakout of exclusively the AD cases from Subtype 6 and Subtype 3, comparing levels of the two AD pathology associated modules, M5: Glycolysis, and M4: Ubiquitination. While there is a significant difference in M5 levels in AD cases between the two subtypes, there is no significant difference in M4 levels (as assessed by 1-way ANOVA).

Supplemental Figure 8: Impact of Human Serum Albumin (HSA) Dilution on Network Module Proteins and Phosphorylated Tau Levels in AD-like Subtype 6 CSF. (A) DIA-MS analysis of HSA doping was run in parallel to plasma doping experiments. Proteins that overlapped with the top 30 network hub proteins used to subtype the Emory Cohort were broken out by module, and plotted across increasing amounts of HSA, normalized as a percentage of their abundance in pooled Subtype 6. Bold black lines represent the average percent decrease across the subtyping hub proteins for each module. Significance was determined by Tukey adjusted 1-way rm ANOVA comparing initial and final concentrations in each module. **(B)** Levels of phosphorylated tau ($pTau_{181}$, $pTau_{217}$, and $pTau_{231}$) present in pooled Subtype 6 CSF were analyzed by Alamar immunoassay following a 24-hour incubation, with increasing concentrations of HSA (by volume: 0.001%, 0.01%, 0.1%, 1%, 2%). HSA addition did not significantly decrease endogenous levels of any phosphorylated tau species. Significance was assessed for the final concentration by 1-way ANOVA; (*p≤0.05; **p≤0.01; ***p≤0.001, ****p≤0.0001).

bioRxiv preprint doi: https://doi.org/10.1101/2025.03.14.643332; this version posted March 15, 2025. The copyright holder for this preprint (which was not certified by peer review) is the author/funder, who has granted bioRxiv a license to display the preprint in perpetuity. It is made available under aCC-BY 4.0 International license.

Supplementary Figure 1

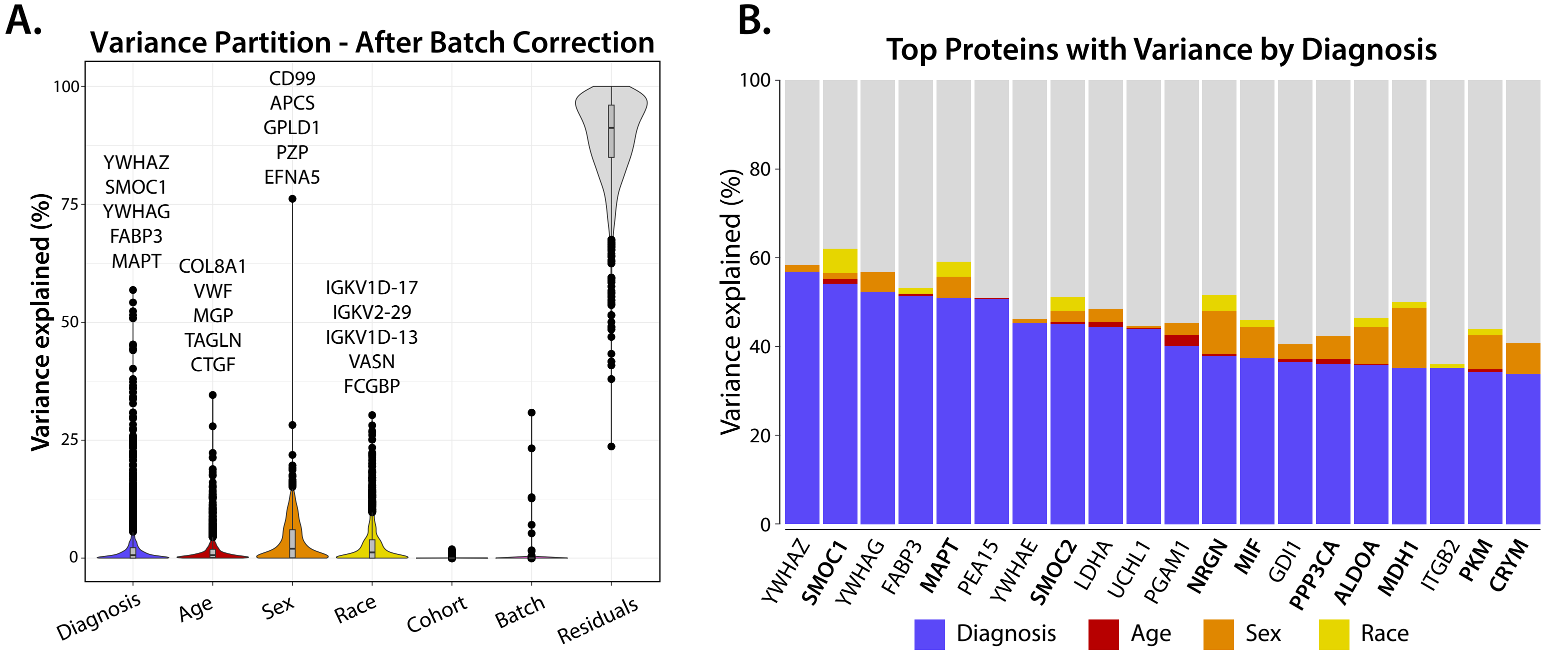

bioRxiv preprint doi: https://doi.org/10.1101/2025.03.14.643332; this version posted March 15, 2025. The copyright holder for this preprint (which was not certified by peer review) is the author/funder, who has granted bioRxiv a license to display the preprint in perpetuity. It is made available under aCC-BY 4.0 International license.

Supplementary Figure 2

A.

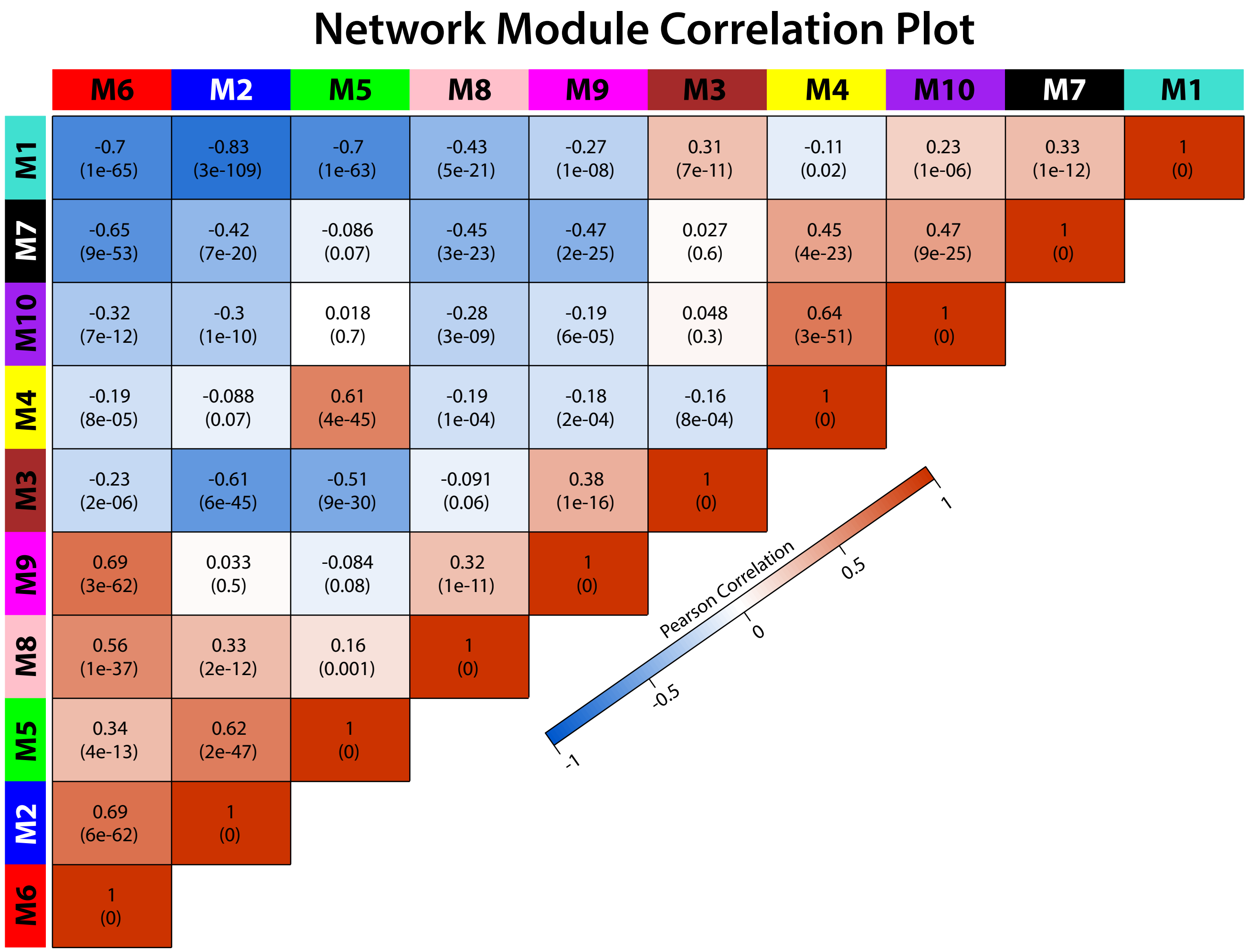

	M6	M2	M5	M8	M9	M3	M4	M10	M7	M1
M1	-0.7 (1e-65)	-0.83 (3e-109)	-0.7 (1e-63)	-0.43 (5e-21)	-0.27 (1e-08)	0.31 (7e-11)	-0.11 (0.02)	0.23 (1e-06)	0.33 (1e-12)	1 (0)
M7	-0.65 (9e-53)	-0.42 (7e-20)	-0.086 (0.07)	-0.45 (3e-23)	-0.47 (2e-25)	0.027 (0.6)	0.45 (4e-23)	0.47 (9e-25)	1 (0)	
M10	-0.32 (7e-12)	-0.3 (1e-10)	0.018 (0.7)	-0.28 (3e-09)	-0.19 (6e-05)	0.048 (0.3)	0.64 (3e-51)	1 (0)		
M4	-0.19 (8e-05)	-0.088 (0.07)	0.61 (4e-45)	-0.19 (1e-04)	-0.18 (2e-04)	-0.16 (8e-04)	1 (0)			
M3	-0.23 (2e-06)	-0.61 (6e-45)	-0.51 (9e-30)	-0.091 (0.06)	0.38 (1e-16)	1 (0)				
M9	0.69 (3e-62)	0.033 (0.5)	-0.084 (0.08)	0.32 (1e-11)	1 (0)					
M8	0.56 (1e-37)	0.33 (2e-12)	0.16 (0.001)	1 (0)						
M5	0.34 (4e-13)	0.62 (2e-47)	1 (0)							
M2	0.69 (6e-62)	1 (0)								
M6	1 (0)									

B.

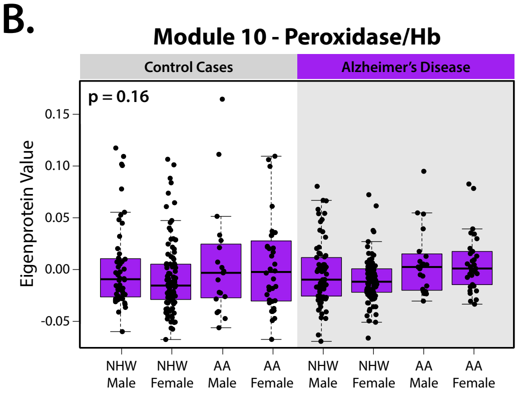

bioRxiv preprint doi: https://doi.org/10.1101/2025.03.14.643332; this version posted March 15, 2025. The copyright holder for this preprint (which was not certified by peer review) is the author/funder, who has granted bioRxiv a license to display the preprint in perpetuity. It is made available under aCC-BY 4.0 International license.

Supplementary Figure 3

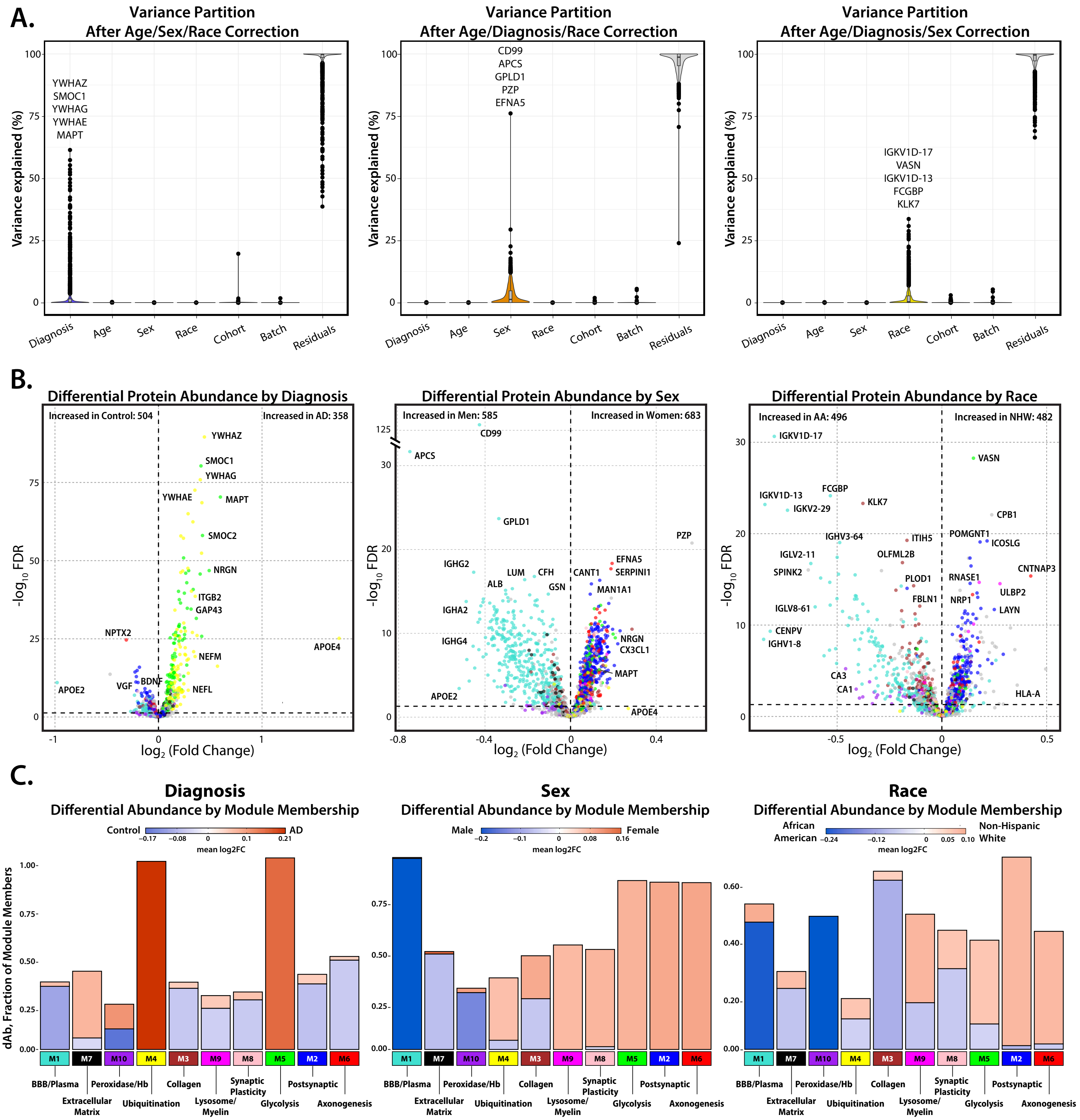

bioRxiv preprint doi: https://doi.org/10.1101/2025.03.14.643332; this version posted March 15, 2025. The copyright holder for this preprint (which was not certified by peer review) is the author/funder, who has granted bioRxiv a license to display the preprint in perpetuity. It is made available under aCC-BY 4.0 International license.

Supplementary Figure 4

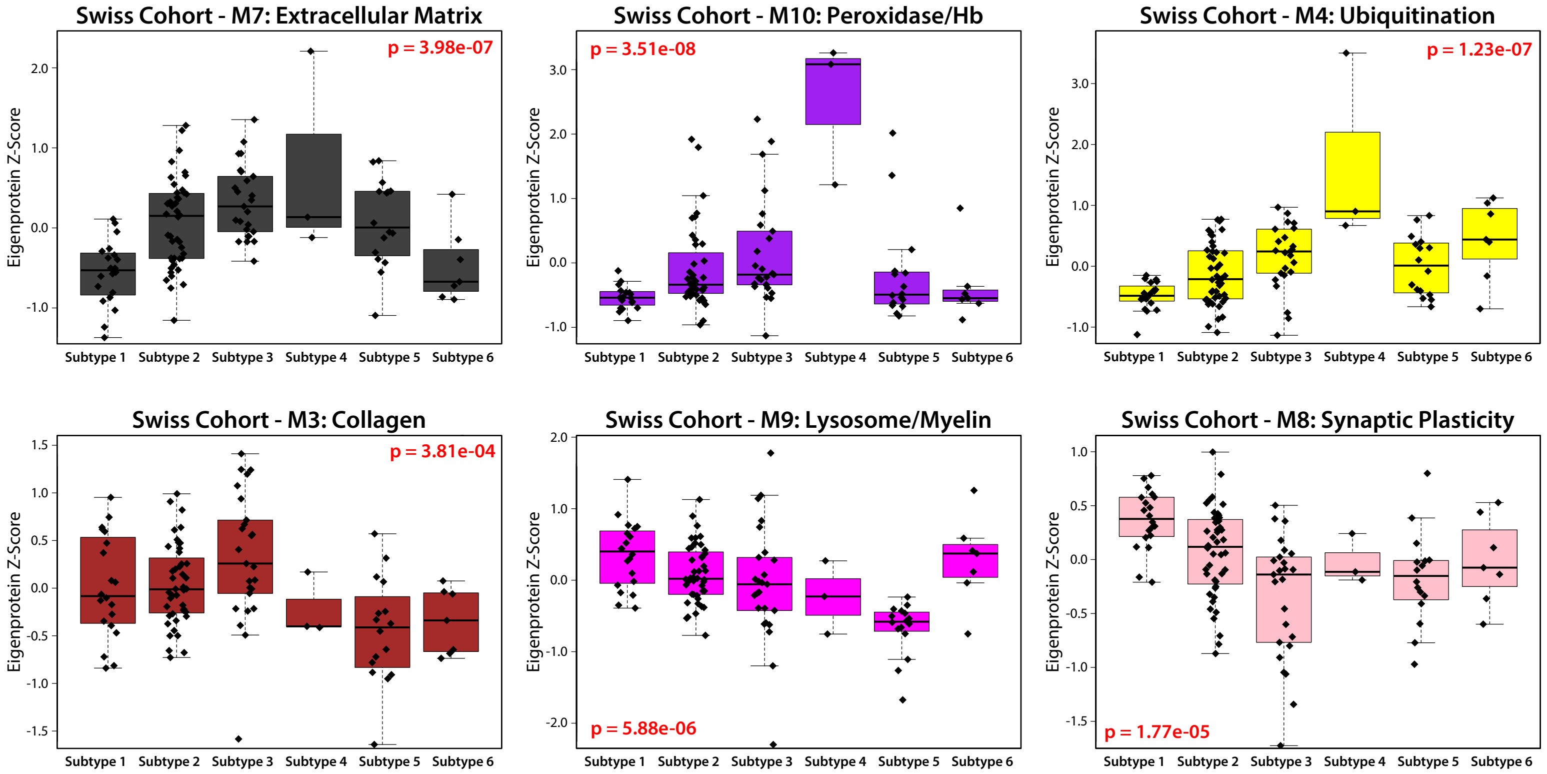

bioRxiv preprint doi: https://doi.org/10.1101/2025.03.14.643332; this version posted March 15, 2025. The copyright holder for this preprint (which was not certified by peer review) is the author/funder, who has granted bioRxiv a license to display the preprint in perpetuity. It is made available under aCC-BY 4.0 International license.

Supplementary Figure 5

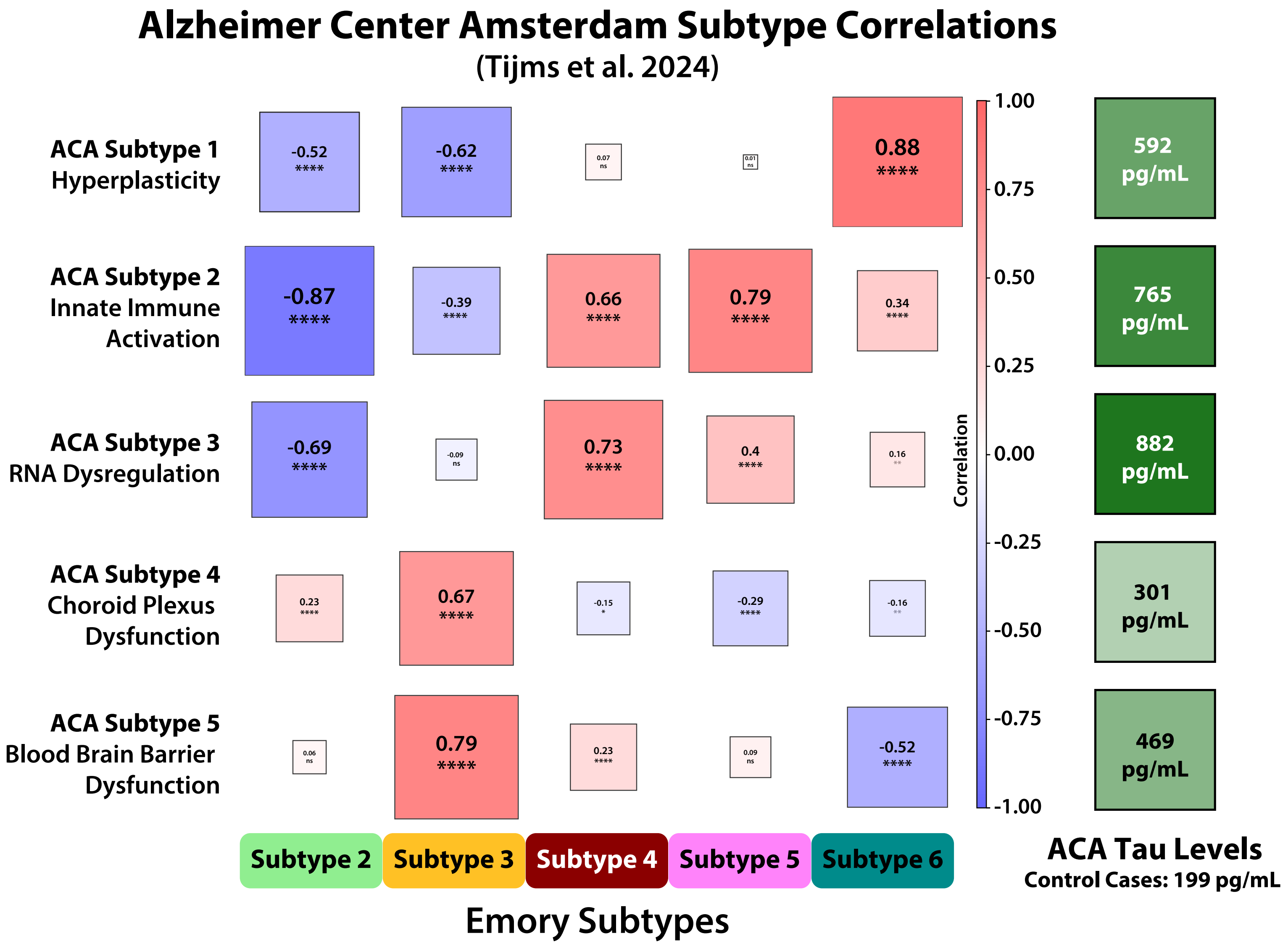

bioRxiv preprint doi: https://doi.org/10.1101/2025.03.14.643332; this version posted March 15, 2025. The copyright holder for this preprint (which was not certified by peer review) is the author/funder, who has granted bioRxiv a license to display the preprint in perpetuity. It is made available under aCC-BY 4.0 International license.

Supplementary Figure 6

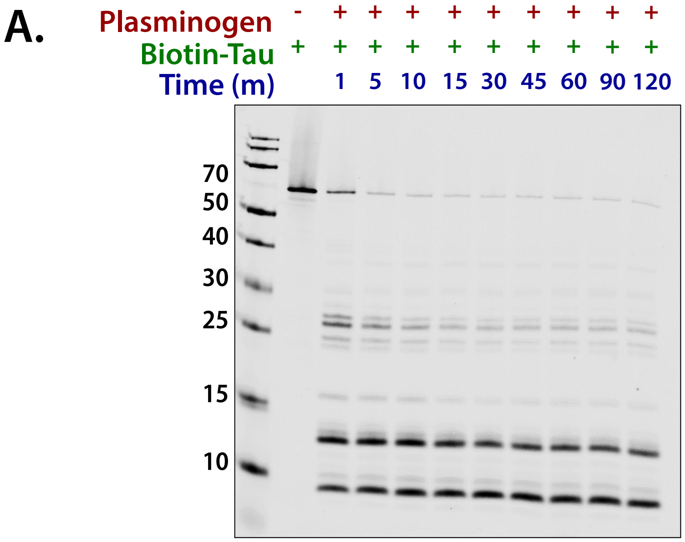

Plasminogen Digestion of Biotin-Tau

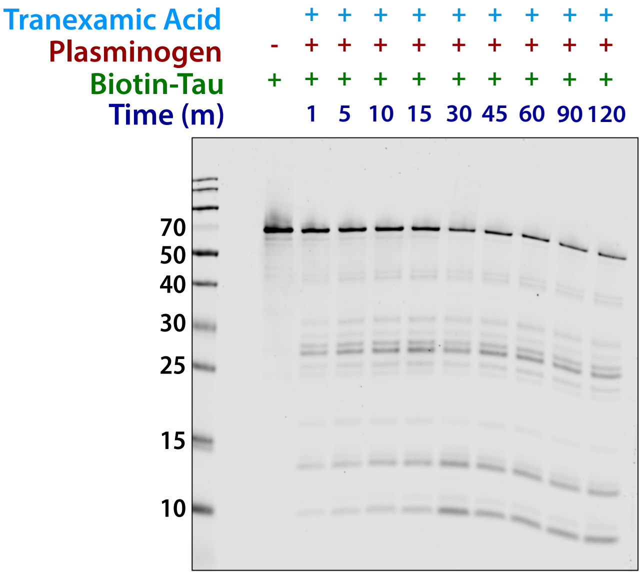

Plasminogen Digestion of Biotin-Tau
With Tranexamic Acid Inhibitor

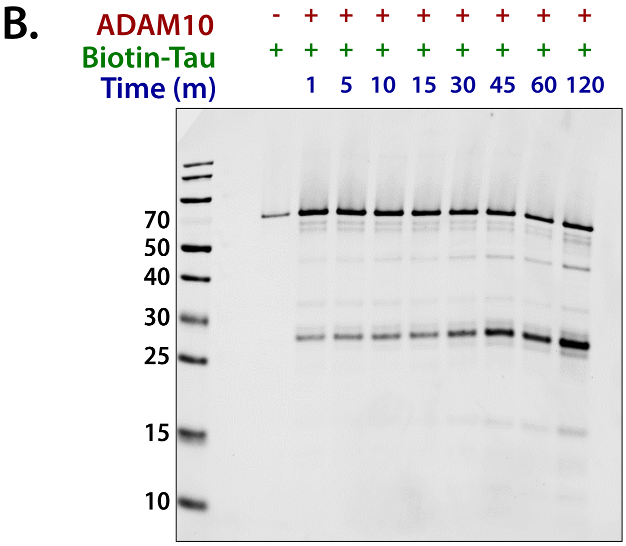

ADAM10 Digestion of Biotin-Tau

bioRxiv preprint doi: https://doi.org/10.1101/2025.03.14.643332; this version posted March 15, 2025. The copyright holder for this preprint (which was not certified by peer review) is the author/funder, who has granted bioRxiv a license to display the preprint in perpetuity. It is made available under aCC-BY 4.0 International license.

Supplementary Figure 7

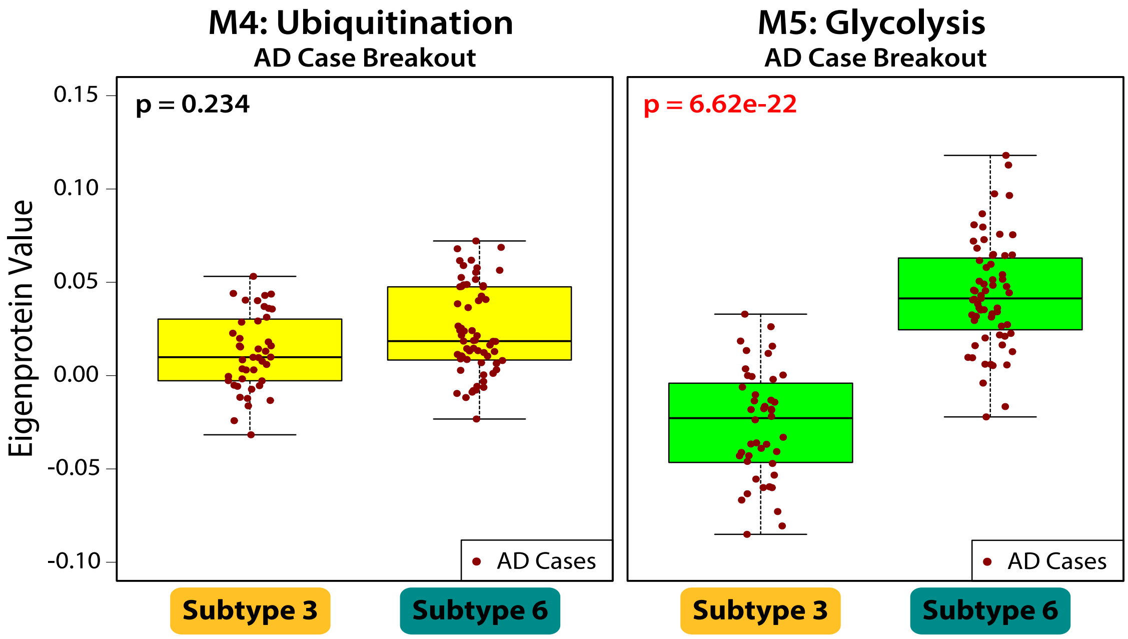

bioRxiv preprint doi: https://doi.org/10.1101/2025.03.14.643332; this version posted March 15, 2025. The copyright holder for this preprint (which was not certified by peer review) is the author/funder, who has granted bioRxiv a license to display the preprint in perpetuity. It is made available under aCC-BY 4.0 International license.

Supplementary Figure 8

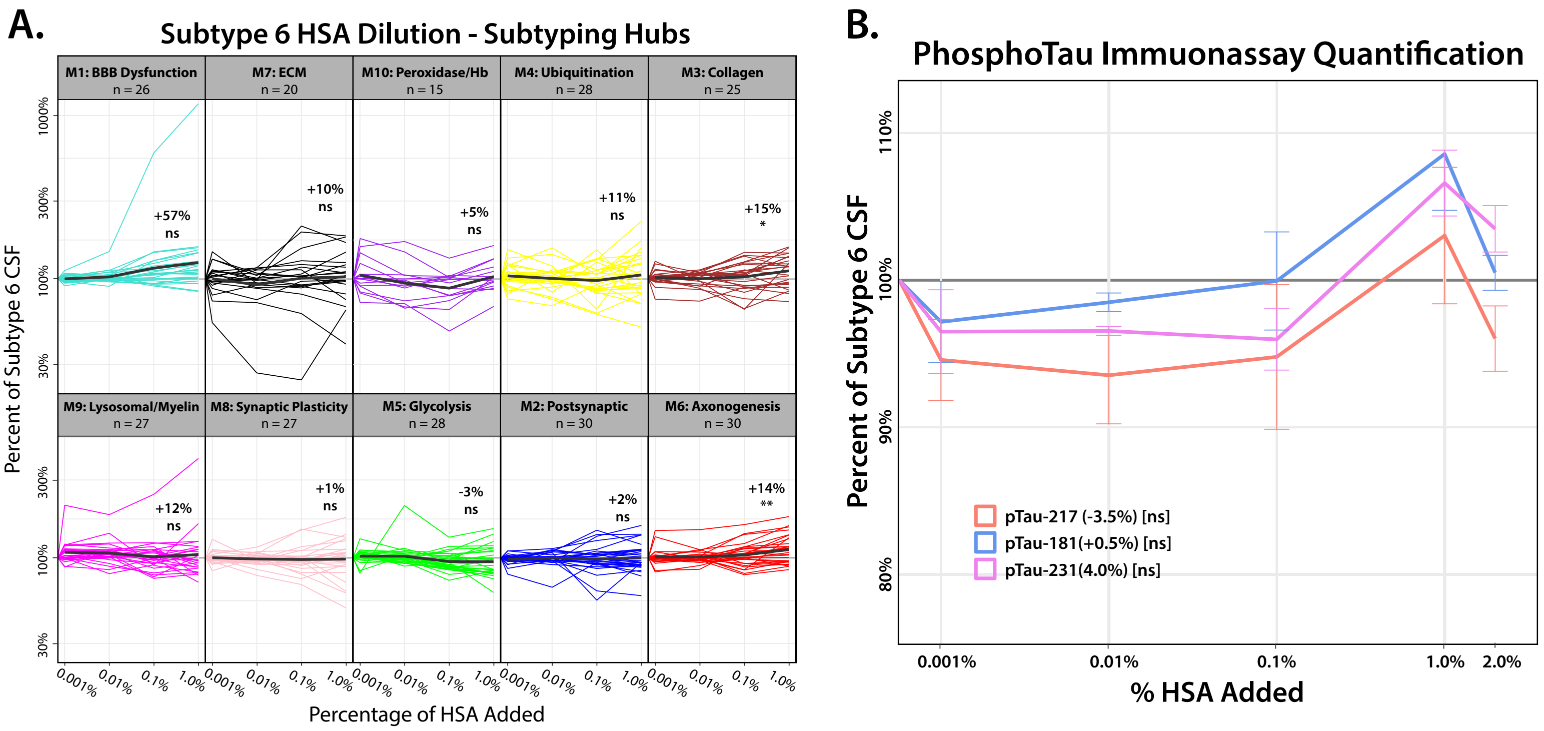

Supplement: 1 [file NIHPP2025.03.14.643332V1-supplement-1.pdf]
